# Facilitators and Inhibitors of Lifestyle Modification and Maintenance of KOREAN Postmenopausal Women: Revealing Conversations from FOCUS Group Interview

**DOI:** 10.3390/ijerph17218178

**Published:** 2020-11-05

**Authors:** Hye-Ryoung Kim, Hwa-Mi Yang

**Affiliations:** College of Nursing, Shinhan University, Dongducheon 11340, Korea; hrkim@shinhan.ac.kr

**Keywords:** health promotion, lifestyle, healthy habits, active life, qualitative research, period, postmenopausal

## Abstract

Modifiable lifestyle behaviors, such as lack of physical activity, smoking, and unhealthy diet, are associated with the risk of cardiovascular diseases in postmenopausal women, in addition to other risk factors, such as aging and physiological changes. Therefore, it is necessary to emphasize the importance of encouraging healthy lifestyles and health-promoting behaviors among postmenopausal women, to achieve a better health status. However, it is difficult to modify lifestyle and maintain that change. This study was aimed at identifying the factors that affect the maintenance of healthy lifestyle habits in postmenopausal women, using various theoretical models. This qualitative study included focus-group interviews with 21 Korean postmenopausal women aged 54 to 69 years. A theory-guided thematic analysis was performed based on the Health Belief Model, Self-Determination Theory, Social Cognitive Theory, and Theory of Planned Behavior. As a result, facilitators and inhibitors to healthy lifestyle modification and maintenance are identified. Various sources of motivation and reinforcement are important for menopausal women to maintain a healthy lifestyle. Autonomy support and self-regulation strategies play an important role in integrating health-promoting behaviors into a daily routine. In addition to personal effort, a social support system is also important to help individuals maintain a healthy lifestyle

## 1. Introduction

Middle-aged women undergo menopause, the natural process that stops the production of female sex hormones due to the aging of the ovaries, causing women to undergo several physical and psychological changes. Up to 80% of menopausal women experience vasomotor symptoms, such as hot flashes and night sweats [1]. Sleep disturbance, depressive symptoms and anxiety, and cognitive performance decline, as well as genitourinary and sexual functioning decline, are also concerns of menopausal changes [1]. Menopausal women are also likely to experience various problems if they do not take proper care of their health. 

Traditionally, Korean patriarchal culture has emphasized family lineage and women’s roles as mothers and wives. Middle-aged Korean women who had been through these cultural climates are urged to sacrifice for their families [2]. As women, in the cultural context of taking care of their family rather than themselves, middle-aged Korean women tend not to care about their own health or well-being until their children become independent and until menopause. The various changes experienced during menopause and the reduced obligation to families may provide opportunities for postmenopausal women in South Korea to focus more on themselves and care about their health.

Postmenopausal women may be especially susceptible to cardiovascular diseases since estrogen withdrawal negatively affects cardiovascular health and metabolism [3]. Cardiovascular diseases, a major health problem, is one of the most important causes of death among postmenopausal women worldwide [4] and is closely associated with obesity in women of this age group. Central obesity, referring to an excessive accumulation of fat in the abdominal area, is reported to increase the risk of cardiovascular diseases [5].

Risk factors for cardiovascular disease in postmenopausal women can be classified as modifiable and not. Even though aging and postmenopausal physiological changes are inevitable, lifestyle behaviors such as smoking, unhealthy diet, and lack of physical activity are adjustable [6,7,8]. Therefore, it is necessary to emphasize the importance of encouraging healthy lifestyles and health-promoting behaviors among postmenopausal women to support them in achieving a better health status. Lifestyle modification and maintenance for health can reduce the risk of diseases, thereby satisfying one’s need to improve health, reduce the medical costs related to disease management, and improve their overall quality of life [9,10].

Many studies have mentioned the need for lifestyle improvements for postmenopausal women to promote better health [8,11]. Several interventional studies have attempted to improve lifestyles by promoting a healthy diet and increasing physical activities, using various approaches [9,11,12], demonstrating improvements in short-term health outcomes resulting from lifestyle improvements. However, other studies have reported that it is difficult to maintain a healthy lifestyle and unlikely for the effect of an intervention on health to continue after the intervention completion [13,14].

Therefore, it is necessary to examine the factors that affect healthy lifestyle habits and allow one to maintain those habits to promote the ability of postmenopausal women to engage in health-promoting behaviors consistently. 

The most common theories in healthy behavior change and maintenance of middle-aged women are Health Belief Model (HBM), Self-Determination Theory (SDT), Theory of Planned Behavior (TPB), and Social Cognitive Theory (SCT) [12,15,16,17]. HBM hypothesizes that health behaviors rely on several factors, such as perceived severity, perceived barriers, perceived benefits, and cue to action [18]. SDT identifies intrinsic motivation as most crucial in initiating and maintaining activities, which are facilitated by autonomy supports. The factors related to autonomy are efforts, enjoyment/interest, value/ usefulness, perceived competence, preference/choice, relatedness, and pressure/tension [19].

TPB considers that motivation and ability are both influences of behavioral achievement. According to TPB Subjective norm, the belief that significant others or groups of people would approve or support a particular behavior is an important factor in motivation. In addition, perceived behavioral control is a key component of perceptions of one’s ability to perform a particular behavior [20]. SCT explains health behavior by using constructs like outcome expectations, observational learning, and self-efficacy [21].

Self-efficacy has been widely studied, with research suggesting that it is a strong predictor of healthy lifestyle modifications [22,23,24]. However, most studies have provided fragmentary evidence with data from structured questionnaires. Moreover, while some researchers have attempted to gain an integrated understanding, they have mostly focused on weight loss or weight maintenance [25] and have been limited to overweight or obese women or adults with health issues [26].

Studies conducted to date have been limited in the comprehensive analysis of the factors that affect the maintenance of health-promoting behaviors in postmenopausal women. Thus, this study was aimed at identifying the factors that affect the maintenance of healthy lifestyle habits in postmenopausal women through focus-group interviews (FGIs) and comprehensively analyzing those factors, using various theoretical models on behavior change and maintenance. The purpose of the study was to provide information that may be used to develop intervention strategies promoting the maintenance of healthy lifestyle modifications in postmenopausal women. 

## 2. Materials and Methods

### 2.1. Design and Participants

We conducted a qualitative study, using FGIs, with 21 Korean postmenopausal women aged 54 to 69 years. All the women had previously participated in a healthy lifestyle modification intervention program at a local community setting. The Healthy Lifestyle Modification Intervention Program was a self-directed, three-month program that allows postmenopausal women to promote health through lifestyle modification and maintenance that emphasizes a healthy diet and increased physical activity. During the intervention program, problematic lifestyle habits were identified, analyzed, and corrected through the process of assessment, diagnosis, planning, execution, and evaluation to promote health behaviors. 

All the participants understood the purpose and methods of this study and provided their informed consent. Thirty-nine participants were initially recruited through phone calls, and the response rate was 53.8%. We carefully selected the participants after considering their age, postmenopausal period, socioeconomic class, educational level, occupational classification, and body mass index. Besides, we considered whether sufficient data on influencing factors for healthy lifestyle modification and maintenance, such as a healthy diet and increased physical activity, could be collected from them, to ensure the high transferability of the results to middle-aged women. Inclusion and exclusion criteria for the study were as follows: (1) at least one year has passed since the last menstruation; (2) those who participated in a healthy lifestyle modification intervention program for over 3 months; (3) those who have voluntary intention to participate in the study; (3) those who are not under the hormonal treatments; (4) absence of psychiatric problems; and (5) no history of strokes, acute myocardial infarctions, or malignant tumors.

### 2.2. Data Collection

Three FGIs were conducted from August 2019 to September 2019. The total of 21 participants was divided into groups of 6–8 for the FGIs. We considered data saturation was achieved, as no newer ideas or themes appeared. Interview dates and time were set at the participants’ convenience. The participants were informed that the interviews would be recorded prior to the interviews. The interviews were conducted in a seminar room with a quiet and comfortable atmosphere. Each interview took approximately 1~1.5 h. The entire interviews were recorded by using three portable audio recorders, to avoid the omission of the interview content. A moderator and co-moderator wrote down their general observations of verbal and nonverbal interactions during the FGIs. All researchers participated in debriefing immediately after the FGI. Thus, interview recordings, observation notes taken during the interviews, and debriefing data obtained after the interviews were collected. 

After clarifying the research problem, the research team developed a rough draft of questions to use in FGIs with the participants. The team then reviewed the rough draft and finalized the questions after a discussion. The questions consisted of an introductory question, transition question, main question, and concluding question. The following questions were used:

- Introductory (5 min): “How have you been doing since you completed the program?”
- Transition (5 min): “What did it mean to you to maintain healthy lifestyles during your participation in the program or your daily life after the program was over?”
- Main (45 min): “Please talk freely about any factors that helped or hindered you from applying and maintaining healthy lifestyle modifications in daily life”.
- Concluding (5 min): “Here is what you have told me so far. Is there anything missing that I should include? Is there anything else you would like to talk about?”

### 2.3. Analysis

Data were analyzed following the four stages of data analysis in Krueger’s (1998) framework. During the first stage, at the start of the research, tasks were distributed among the research team members, and interview questions were derived in preparation for the FGIs. In the second stage, all researchers participated in the FGIs to audio record the interviews and took notes of their observations. Third stage, immediately after each FGI, was a debriefing wherein, the researchers reviewed and shared a summary of their interview notes. In the fourth stage, the interview recordings were transcribed, to complete the data preparation process for analysis. 

Theory-guided thematic analysis was performed based on the HBM, SDT, SCT, and TPB. Before analysis, the researchers defined every construct of theories, and then they repeatedly read the interview transcript to understand its content fully. We read each sentence in the transcript one by one and included it in a summary if we all agreed the sentence was meaningful. The relevant meaningful parts of the recorded sentences were then turned into code and mapped to constructs of the theories. In the next stage, tentative subthemes were derived from the codes, and data were collected on these subthemes. Themes were derived by grouping similar subthemes together. After a review, the subthemes and themes were assigned names and defined for differentiation. To ensure research rigor, the researchers independently analyzed the data and reached a consensus on the analysis results over several discussions. In cases where an agreement was not reached, the researchers asked the participants to clarify their statements. To eliminate biases and ensure data confirmability, all steps of the analysis were reviewed by an expert experienced in conducting qualitative research on behavioral change. 

### 2.4. Ethical Consideration

This study was approved by the Institutional Review Board of Shinhan University (SHIRB-201806-HR-078-02), to ensure the research methodology and procedures abided by research ethics. The participants were sufficiently informed about the methods, purpose of this study, and the confidentiality. Only the participants who expressed voluntary consent to participate in the study were included. All data were anonymous.

## 3. Results

### 3.1. Participant Characteristics

The study participants were residents of Seoul and suburbs in Korea, with an average age of 58.6 years and an average postmenopausal period of 7.6 years. Participant characteristics, such as socioeconomic level, education level, job classification, and degree of obesity, are summarized in Table 1.

### 3.2. Codes, Themes, and Subthemes

**Figure 1:**
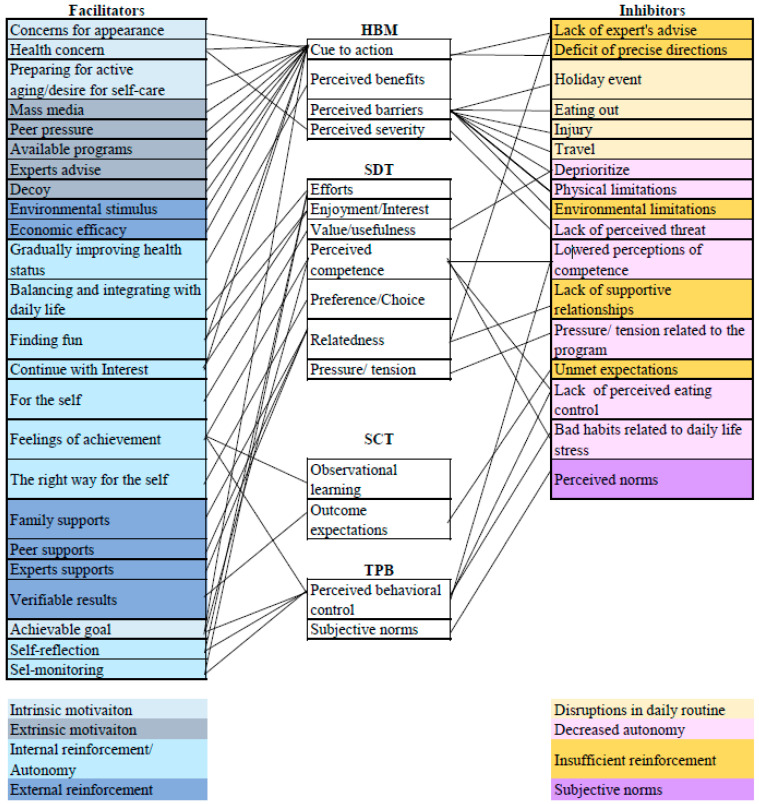
Theory guided mapping. HBM: health belief model; SDT: Self-determination theory; SCT: Social cognitive theory; TPB: Theory of planned behavoir.

We identified 24 open codes for the factors promoting the maintenance of healthy lifestyle modifications and 17 for the factors hindering the maintenance of healthy lifestyle modifications in postmenopausal women. The constructs of theories that match each code are shown in Figure 1.

The facilitators of healthy behavior maintenance could be categorized into four subthemes: “Intrinsic cue to action”, “Extrinsic cue to action”, “Internal reinforcement/Autonomy”, and “External reinforcement”. Two themes were derived from the four subthemes: “Taking a step toward healthy behavior maintenance using intrinsic and extrinsic motivational factors” and “Lifestyle rebalancing under the self-regulation employing internal and external reinforcement” (Table 2).

The inhibitors to healthy behavior maintenance could be categorized into the following subthemes: “Disruptions in daily routine”, “Decreased autonomy”, “Insufficient reinforcement”, and “Subjective norms”. The following themes were derived from these subthemes: “Failure to integrate the healthy habits into lifestyle” and “Inappropriate supportive strategy” (Table 3).

#### 3.2.1. Taking a Step toward Healthy Behavior Maintenance Using Intrinsic and Extrinsic Motivational Factors

Intrinsic and extrinsic motivations for lifestyle modification were found to promote the maintenance of a healthy lifestyle in postmenopausal women. 

Four sources of intrinsic motivation were identified: concerns about appearance, health concerns, preparing for active aging/desire for self-care, and achievable goal.

*I worked out because I was desperate. I have to wear tight clothes when riding a bike. That way, when I see myself, I could see how my fat bulges out on my front and back (FGI1-PM52807).*

Based on statements such as the one given above, appearance concerns were identified as a factor promoting the maintenance of a healthy lifestyle and could be considered a cue to action in the HBM. The following statement also confirmed that appearance concerns resulting from the weight gain that consistently occurs after menopause promoted the maintenance of a healthy lifestyle:

*I reached menopause when I was 56. My weight gradually and continuously increased from that time on. Day after day, I would keep breaking the record. I felt quite miserable as I could no longer fit into nice clothes (FGI2-PM52808).*

The following statements showed that health concerns acted not only as a cue to action but also as perceived severity in the HBM in postmenopausal women, thereby becoming an intrinsic motivational factor.

*I think the desperate desire to get healthy after falling sick is more important. I found myself working harder to maintain a healthy lifestyle after I became sick (FGI3-PM51903).*

*This time, I felt the desperate need to not gain any more weight and not let my sugar levels rise since my weight had been continuously increasing. I worked out very hard after I started participating in this program. I began to lose weight as a result… (FGI2-PM52404)*

Preparing for active aging/desire for self-care was also identified as an intrinsic motivational factor among postmenopausal women since they are at a stage in life where they are preparing for old age.

*We are aware that whereas people in our generation take care of our parents, people in the current generation would never do such a thing. We need to stay healthy to be able to take care of ourselves instead of being taken care of by our children. The idea that I need to take care of my own health seems to motivate me (FGI4-PM52305).*

Traditionally in Korean society, caring for older parents was taken for granted as a child’s duty, but sociocultural changes may lead middle-aged women to accept the idea that they have to take care of their own health. In particular, in the above sentence, it can be confirmed that the recognition that maintaining health is preparing for active aging in terms of securing independence of activities acts as a motive for maintaining a healthy lifestyle for postmenopausal women.

The following statement also confirmed that preparing for active aging/desire for self-care are intrinsic motivational factors for health-promoting behaviors.

*Whenever I eat, I would tell myself it’s not right as a parent to make my children feel worried. I’ve been trying to resist unhealthy food and eat something healthier (FGI3-PM52601).*

Setting a realistic goal based on previous experience with healthy lifestyle modifications and behavioral changes were identified as an intrinsic motivational factor for the maintenance of a healthy lifestyle and could be considered perceived competence, a determinant of autonomous motivation, in the SDT, perceived behavioral control in the TBP, and a cue to action in the HBM.

*I think it would be easy if I have a goal. I once got rid of hypertension by exercising. I started participating in the program hoping to reduce my medication use if not completely stop it, with that goal in mind… or the goal to lose weight… I don’t want to regain the weight that I lost (FGI3-PM51903).*

Mass media, peer pressure, available programs, experts’ advice, and decoys were identified as the extrinsic motivational factors for the maintenance of a healthy lifestyle in postmenopausal women (Table 1). These motivational factors could be considered as a cue to action in the HBM. 

#### 3.2.2. Lifestyle Rebalancing under the Self-Regulation Employing Internal and External Reinforcement

Modifying a lifestyle by self-regulation with external and internal reinforcement promoted the maintenance of a healthy lifestyle in postmenopausal women. Internal reinforcement could be considered as perceived benefits in the HBM, various determinants of autonomy in the SDT, perceived behavioral control in the TPB, and observational learning in the SCT.

The determinants of autonomy in the SDT functioned as a source of internal reinforcement to help postmenopausal women maintain health-promoting behaviors. Having an interest in health-promoting behaviors and deriving fun from engaging in them provides internal reinforcement that promotes the maintenance of health-promoting behaviors.

*Even on a hot summer day, even when it was raining, I made sure to walk on Wednesdays since Wednesdays were set as the “Walking Day”. On rainy days, the three of us would be walking in white raincoats (laughing). It was fun too. Walking was an extra source of fun aside from volunteering on Wednesdays. I had a lot of fun losing weight this summer (FGI2-PM52905).*

*The reason why I am extending my participation is that I determined to increase my muscle mass for the next three months. I got curious about how much effort would be needed to gain how much muscle mass. Now that I can check my muscle mass on my own… (FGI2-PM52404).*

Furthermore, realizing that health-promoting behaviors are for one’s benefits and choosing health-promoting behaviors according to one’s preference also promoted the maintenance of health-promoting behaviors.

*It’s for me… It’s not for others. Even if I feel lazy, I still need to do it because it’s for myself. I think I did it just as how we live through our daily life (FGI4-PM53001).*

*I enjoy walking, so walking seems to work best for me. Everyone has a different method that works for them. To me, it was walking (FGI4-PM53001).*

Perceived behavioral control and feeling a sense of achievement from observational learning also promoted the maintenance of health-promoting behaviors.

*I would exercise early in the morning, but some days, I feel tempted to just go home and sleep more. But once I beat that temptation and come back home after completing my exercise, I can start my day in a good mood (FGI2- PM52905).*

*I started out by walking. I noticed others were also walking. Once, a young person was jogging not too fast like this, and I tried to do the same, and it worked (FGI4- PM51602).*

Health-promoting behaviors must be incorporated into daily life in a balanced manner to be consistently maintained. The effort, a determinant of autonomous decision-making, is associated with the process of integrating health-promoting behaviors into a lifestyle.

*I wouldn’t call what I did an exercise, but whenever I had some free time during daily life, or there was sufficient time between one schedule and another, I would walk instead of taking the subway. I would walk around an hour this way (FGI1-PM52807).*

*I forced myself to do what I didn’t want to do and walked 10,000 steps once per week. I tried to walk over 10,000 steps within a week or walk at least one hour per day. Although I am doing the same thing over and over, it is hard to keep the habit of walking. I think being consistent is challenging. But over time, I slowly got used to walking (FGI2-PM52405).*

A self-regulatory strategy such as self-monitoring and self-reflection promoted the maintenance of health-promoting behaviors and is associated with perceived behavioral control in the TBP.

*Since I needed to fill out the checklist… I didn’t want to leave too many checkboxes empty, so I tried my best (FGI1-PM52801).*

Postmenopausal women tend to accept the idea that weight gain is natural and inevitable because of aging and decreased metabolic changes. However, when they recognize their modifiable problematic lifestyle through self-reflection, these perceptions may lead to motivation to change their lifestyle, as stated below.

*I already knew that my lifestyle was blamed to be fat. But when I checked one by one in this program, it seems clear that I was getting fat because of things that I should have changed (FGI2-PM52405.)*

Seeing gradual improvements in one’s health also promoted the maintenance of health-promoting behaviors via internal reinforcement.

*Seeing numerical improvements helped me understand my progress and motivated me to work harder (FGI4-PM53001).*

Supports from family, peers, and experts, environmental stimulus, economic efficacy, and verifiable results were identified as the external reinforcement factors for maintaining health-promoting behaviors in postmenopausal women (Table 1). 

The following statement demonstrates that verifiable results fall under the outcome expectation construct of the SCT.

*Since I knew precise results would come out after a few months, I worked harder to achieve better results. When there are no accurate data to show your progress, you tend to slack off even if you were determined in the beginning. Now, I think about how my cholesterol levels would change in a blood test a few months later and am careful about what I eat (FGI2-PM52808).*

#### 3.2.3. Failure to Integrate Healthy Habits into a Lifestyle

Based on the results of this study, postmenopausal women who failed to integrate healthy lifestyle habits into their daily lives due to disruptions to their daily routines and decreased autonomy found it difficult to maintain health-promoting behaviors. 

Holiday events, eating out, injury, and traveling were instances where participants broke their daily routines. These situations could be considered perceived barriers in the HBM. 

Decreased autonomy could be explained as a lack of perceived barriers and perceived severity, which are constructs of the HBM, problems in the determinants of autonomy in the SDT, and problems with perceived behavioral control in the TBP. In this study, perceived barriers associated with autonomy in postmenopausal women included reduced physical strength and physical limitations such as decreased energy level. Participants were aware of the importance of health-promoting behaviors but prioritized them behind other tasks and failed to maintain them, as demonstrated in the following statement:

*My thyroid conditions make me feel weak and lethargic. I keep skipping exercises by making excuses about my thyroid conditions. It’s actually difficult for me to move. You know what I mean… I feel very tired and crave sweets. I gained two kilograms as a result, and it’s been hard to lose the gained weight (FGI3-PM52805).*

*I do think that I need to work out, but the busy schedule I set for myself keeps me from working out (FGI3-PM52303).*

Not feeling a sense of crisis about one’s health can also lead to a lack of severity perception and subsequently, reduced autonomy, which interferes with women’s ability to maintain health-promoting behaviors. Table 2 presents statements demonstrating this phenomenon. Furthermore, having a negative perception of their ability to maintain health-promoting behaviors also reduced the women’s autonomy and hindered the integration of healthy habits in their daily lives. The negative perception could be explained by a lack of competence, one of the determinants of autonomy in the SDT.

*I feel so pathetic and depressed. I would try but failed. I would see how weak-willed I am and feel unenergetic. And then again, I would tell myself to try again. Even then, I’d still feel unenergetic for a long time. I’m so pathetic (FGI4-PM51602).*

The following statements demonstrated that competence is affected even when someone feels that they have no perceived behavioral control, which is a TPB construct.

*I love sweets. My head tells me not to eat them, but once I see them, I can’t help it. I think that’s the problem (FGI4-PM52004).*

*I spend a lot of my time with kids, and I love eating. I can’t help but eat some of my kids’ snacks… I think I felt stressed about having my alone time taken away by the kids and tried to cope with the stress by eating, which resulted in my weight gain (FGI2-PM52905).*

The following statement demonstrated that excessive stress can prevent the maintenance of health-promoting behaviors. This can be explained by pressure/tension in the SDT.

*To me, the pressure to keep up was very stressful. I felt like if I didn’t keep up, I would fall behind everyone else. Having to fill out the self-check list on the computer according to the schedule was too much for me, so I just gave up (FGI3-PM61903).*

#### 3.2.4. Inappropriate Supportive Strategy

An appropriate support strategy is needed to maintain a healthy lifestyle, and maintenance of a healthy lifestyle was difficult for postmenopausal women who did not receive sufficient support. 

The subthemes identified as inappropriate support strategies in this study were insufficient reinforcement and subjective norms. Insufficient reinforcement was found to be associated with relatedness, which is a determinant of autonomy in the SDT, outcome expectations in the SCT, and perceived barriers in the HBM (Table 2). 

Lack of advice from an expert or lack of precise instructions regarding health-promoting behaviors were examples of cases where relatedness was not satisfied. Not having someone to help the participants maintain health-promoting behaviors was also a factor that hindered their behavior maintenance.

*I didn’t want to hike alone, so I just didn’t go hiking. My blood pressure started rising again after I stopped hiking (FGI3-PM51903).*

*I had fun exercising during the program, but I couldn’t get myself to exercise alone at home (FGI1-PM52601).*

Korean middle-aged women, who are accustomed to the sociocultural climate that functions as a member of the family rather than presenting themselves as a subjective being, tend to find companions to be with in choosing and continuing health behaviors. This tendency shows that the satisfaction of the desire for relatedness is one of the important factors in healthy behavioral change and maintenance in Korean postmenopausal women.

Additionally, participants whose expectations regarding the outcomes of engaging in health-promoting behaviors were not met found it difficult to continue the health-promoting behaviors, as demonstrated by the following statements.

*You know how you feel when your results do not reach your expectations. When we were young, we would eat a lot but if you exercise one day, you’d see the effect of it the next day. Now, even after three or four days, we don’t see any improvements because we’re old. Since we’re not seeing immediate improvements, it’s harder to maintain a healthy lifestyle. Seeing even just a small improvement would motivate us but without seeing any improvement, we can’t get ourselves to it (FGI4-PM52004).*

*I was hopeful that since doing I was something that I never used to do, I would see drastic improvements, but that was not the case… and it made me skeptical. Since there were no immediate improvements… (FGI4-PM52004).*

Environmental restrictions also functioned as perceived barriers inhibiting the reinforcement of health-promoting behaviors.

*I used to go to the gym to ride a bike machine and run on a treadmill, but the air inside the gym felt so suffocating. I ended up not going to the gym often… (FGI1-PM52801).*

*When it’s raining heavily, I don’t go out. When there’s no one outside, it’s scary. The Han River is bustling with people even after midnight. At the Ttukseom Park, you can go out any time to exercise, but even there, it’s scary when it’s rainy and dark with no people around… (FGI1-PM52101).*

Korean postmenopausal women have a tendency to experience a new vitality in life and the value of work by sublimating loneliness and weakness in relationships with family or others through conversations with peers. Perceptions of friends or social implications in these situations, even if they are negative, have a greater impact on health behavior choices than individuals’ beliefs. The following statements demonstrate the instances where subjective norms hindered the maintenance of health-promoting behaviors among postmenopausal women.

*What I really hate is when a friend brings brown rice to our hangout session because she’s on a diet. I hate that! She shouldn’t have come! Her doing that really breaks the atmosphere. I get that she’s strong-willed and may later be successful with weight loss, but I let myself eat when we’re hanging out. I guess it’s just different opinions, but people like her ruin my mood. How can you bring a lunch like that? I just let myself loose when we’re hanging out and eat to my heart’s content. That’s why I have all this fat (FGI3-PM52306).*

*I felt so weak after I lost around one kilogram. I told my friends I felt weak, and my friends told me, “Hey, it’s because you lost weight. Just eat” (laughing). They told me that “people who eat well” look well and that when you lose weight at our age, you are bound to feel weak (FGI2-PM52808).*

There was a time in Korea when gaining weight was considered a symbol of wealth. This idea also shaped a social perception that it would be nice to gain some weight with age. The above statement reflects these sociocultural influences.

## 4. Discussion

In this study, the facilitators and inhibitors of the maintenance of a healthy lifestyle in postmenopausal women were identified based on various behavior change and maintenance theories. 

Appearance concerns were identified as an internal motivational factor for maintaining health-promoting behavioral changes in this study. Such concerns about postmenopausal weight gain and body shape motivated participants to increase their physical activities and maintain these behavioral changes. Similarly, a study by Barragan et al. (2018) demonstrated that men aimed to lose weight to improve health, whereas women placed higher importance on aesthetics [27]. However, due to the physical and metabolic characteristics of postmenopausal women who expected rapid and dramatic changes by applying healthy lifestyle behaviors, their motives were found to act as a negative factor when these expectations were not met. Therefore, a correct understanding of the physical and metabolic changes in menopause and setting achievable realistic goals is an important strategy for changing and maintaining the healthy lifestyles of postmenopausal women.

Realistic goal setting served as an internally motivating factor for our study participants, too. This finding is consistent with Walker et al.’s (2018) report that a lack of progress and unaccomplished goals reduce the motivation to maintain health-promoting behaviors [28]. Lifestyle change maintenance is highly likely to be reinforced when a person recognizes his or her capabilities by accomplishing an achievable goal [29]. These results indicate that it is necessary to support postmenopausal women by encouraging them to set realistic goals, in a step-by-step manner, to help them maintain health-related behavioral changes. 

Middle-aged women experience physical and psychological changes as they undergo menopause [1]. Decreased physical functioning and metabolic energy decline interfere with active physical activity in postmenopausal women, which can exacerbate health problems [30]. Under these circumstances, postmenopausal women perceive these changes as aging or inevitable changes to some extent and sometimes take a passive attitude. However, through these changes in menopause, Korean middle-aged women are also an opportunity to check their health and prepare for old age, freeing from the attitude that prioritized their family over themselves. Health concerns and preparing for old age were identified as characteristic facilitators of the maintenance of health-promoting behaviors in this study. Active and healthy aging has traditionally focused on preventing chronic disease, but maintaining physical, mental, and cognitive functional status; securing the independence of activities; and participating in and enjoying sociocultural life are more concerning, which is supported by adopting a healthy lifestyle, such as more physical activity and healthy eating [31]. From this point of view, it is necessary to include motivational support for active and healthy aging in a strategy facilitating postmenopausal women to develop and maintain a healthy lifestyle.

External motivational factors of maintaining health-promoting behaviors were mass media, peer pressure, available programs, experts’ advice, and decoys, suggesting that sociocultural support helps individuals maintain healthy lifestyle modifications. The influence of media on one’s health-promoting behaviors has been evidenced through a study that reported exposure to food-related television advertisements can induce obesity [32]. Additionally, Poirier et al. (2016) reported that a partner, friends, and families have substantial influence on a postmenopausal woman’s choice of a healthy diet [33].

However, these factors alone do not sufficiently promote the maintenance of health-promoting behaviors; internal and external reinforcement and appropriate support strategies are also necessary. A systematic literature review on behavioral change theories reported that behavioral reinforcement is more affected by immediate benefits or emotional results than by long-term or logical results [34]. In other words, health-promoting behaviors are maintained when one derives enjoyment and a sense of achievement from the behavior and is aware of its values and when autonomy is supported rather than when external motivation is provided [35]. Behavioral changes are more well-maintained when people are aware that the newly acquired habit matches their values, and that they can choose to engage in new habits on their own accord [35]. Therefore, when planning an intervention to promote health-promoting behaviors in postmenopausal women, it may be necessary to consider strategies such as autonomy support based on the SDT. 

For lifestyle modification maintenance, it is necessary to habituate a newly acquired behavior by making lifestyle adjustments and integrating the behavior into daily life. Lifestyle adjustments occur as one develops healthy lifestyle habits while removing bad ones [35]. Habits are behaviors that automatically occur in response to a situational cue and form when self-regulation of a new behavior successfully occurs over a certain length of period. Habits can be consciously controlled in initially, but as they repeatedly occur over time, they eventually occur automatically, outside of one’s awareness [35]. However, if an old habit is stronger than the newly acquired habit, one tends to regress to the old habit. Therefore, developing an automatic response through habituation and decision-making through self-reflection and self-regulation are important factors of maintaining health-promoting behaviors [36]. Therefore, interventions that promote the maintenance of health-promoting behaviors in postmenopausal women must consider self-regulatory strategies such as self-monitoring and self-reflection. 

Self-regulatory behaviors that occur during the process of integrating a health-promoting behavior into a daily routine and adjusting lifestyle includes making a realistic plan, prioritizing, and engaging in self-reflection and self-monitoring [29]. Environmental restrictions and inadequate support negatively affect self-regulation during this process, consequently hindering the habituation of healthy lifestyle habits. To remove these obstacles, motivational support must include feedback from an expert and instructions on for setting realistic goals and interpreting results [26].

Social awareness affects the opportunity cost-and-reward system associated with a person’s choice of behavior in a given situation [35]. This awareness can affect the amount of effort needed to maintain a healthy lifestyle. When behavior complies with social norms, one’s ability to maintain the behavior increases. However, as demonstrated in this study, if the social norm does not match the health-promoting behavior, it acts as a negative pressure on behavior change, and more efforts are required to overcome this. To reduce this pressure, improving sociocultural awareness of healthy lifestyles and providing social supports may be more effective than providing individual support. 

According to the SDT, meeting relatedness reinforces intrinsic motivation through the support of autonomy. However, for Korean postmenopausal women who are accustomed to the collectivistic culture, it is difficult to overcome social norms even if it negatively affects their health. In this way, autonomy support through satisfying relatedness, the constructs of the SDT does not necessarily lead to positive effects. Therefore, the sociocultural context must be considered when making the strategy of strengthening the relatedness for autonomy support to promote healthy lifestyle modification and maintenance.

This study identified the factors that affect postmenopausal women’s maintenance of health-promoting behaviors based on various behavioral change theories and attempted to make connections between the theories and reality. However, since the research includes participants in specific health-promotion programs, and the participants’ socioeconomic and educational levels are relatively high, care should be taken to generalize the results. Furthermore, the results regarding social norms must be interpreted with care, since societies may vary in their perceptions of a healthy lifestyle depending on their culture. 

## 5. Conclusions

This study examined the facilitators and inhibitors of the maintenance of health-promoting lifestyle modifications in middle-aged women, who undergo drastic physical and psychological changes as a result of menopause, based on various theories. Various theories on health behavior change and maintenance have been developed so far, but there was no best-fit theory applicable to postmenopausal women. Various sources of motivation and reinforcement are important for menopausal women to maintain a healthy lifestyle. Autonomy support and self-regulation strategies play an important role in integrating health-promoting behaviors into a daily routine. In planning an effective intervention program for promoting a healthy lifestyle of postmenopausal women, it is necessary to consider the social and family context within the community not just emphasizing individualized motivation. In addition to personal effort, a social support system is also essential to help individuals maintain a healthy lifestyle. Additional studies with postmenopausal women are needed from various countries, to determine cultural factors of support for maintaining their lifestyle changes at this time in their lives. Moreover, future studies could utilize follow-ups to measure the impacts of support and healthy habits and lifestyle changes on the health of women over a longer period.

## Figures and Tables

**Table 1:** General characteristics of participants.

*N* = 21		
Category	Classification	Mean ± SD or *n* (%)
Age		58.6 ± 4.02
Postmenopausal period (years)		7.6 ± 5.81
Socioeconomic class (self-rated)	High	6 (28.6)
	Middle	13 (61.9)
	Low	2 (9.5)
Education level	University degree or above	13 (61.9)
	High school education	6 (28.6)
	Middle school	2 (9.5)
Occupational classification	Housewife	18 (85.7)
	Office workers	3 (14.3)
BMI (body mass index, kg/m^2^)	Underweight (18.5 or below)	1 (4.8)
	Normal weight (18.5–22.9)	9 (42.9)
	Pre-obese (23.0–24.9)	4 (19.0)
	Overweight (25.0–29.9)	6 (28.5)
	Obese (≥30.0)	1 (4.8)

**Table 2:** Facilitators of healthy lifestyle modification and maintenance.

Facilitators (Participants’ Quotes)	Code	Subtheme	Theme
I worked out because I was desperate. I have to wear tight clothes when riding a bike. That way, when I see myself, I could see how my fat bulges out on my front and back.	Concerns about appearance	Intrinsic motivation	Taking a step toward healthy behavior maintenance using intrinsic and extrinsic motivational factors
I reached menopause when I was 56. My weight gradually and continuously increased from that time on. Day after day, I would keep breaking the record. I felt quite miserable as I could no longer fit into nice clothes.			
I was shocked after measuring my waist circumference. I was so embarrassed. My goal was to reduce my waist circumference, and that was it. I was so embarrassed that day.			
This time, I felt the desperate need to not gain any more weight and not let my sugar levels rise since my weight had been continuously increasing. I worked out very hard after I started participating in this program. I began to lose weight as a result.	Health concern		
I have diabetes... I love sweets, but I didn’t know I had diabetes. My father had diabetes, but it didn’t run in my families. I never thought I would have diabetes, but once, my results showed high sugar levels. I even retook the test to make sure the results were correct since I had no idea I had diabetes. Anyways, I really needed to lose weight. They said my sugar levels can be controlled, and I was in a little bit of disbelief, but since I really needed to do it... since something bad could happen if I didn’t... Yeah... I got very scared.			
I think the desperate desire to get healthy after falling sick is more important. I found myself working harder to maintain a healthy lifestyle after I became sick.			
‘I think it would be easy if I had a goal. I once got rid of hypertension by exercising. I started participating in the program hoping to reduce my medication use if not completely stop it. With that goal in mind… or the goal to lose weight… I don’t want to be fat again.	Achievable goal		
Whenever I eat, I would tell myself it’s not right as a parent to make my children feel worried. I’ve been trying to resist unhealthy food and eat something healthier	Preparing for active aging/desire for self-care		
That could be one of the reasons why I pulled myself together. I think about how I don’t want to become a burden to my children whenever I don’t want to exercise and force myself to dress up and go outside.			
They say, “you need to die well”. I don’t know how long I will live but during what remaining time I have, I want to be healthy and independent and not bother anyone. Whenever I think about this, I get waken up again (laughing). I tell myself I should die well and stay healthy while I’m alive.			
We are aware that whereas people in our generation take care of our parents, people in the current generation would never do such a thing. We need to stay healthy to be able to take care of ourselves instead of being taken care of by our children. The idea that I need to take care of my own health kind of motivated me.			
I don’t really like onions. But once, I made onion pickles after I saw on a television show that onions are good for health. My diet gradually improved unknowingly. When a television show tells you something is good for losing fat, I’d give it a try.	Mass media	Extrinsic motivation	
People tell me I look well because they don’t want to directly tell me that I gained weight. Before, people used to tell me I gained a lot of weight. The first few times, I just responded with “Oh, I see...” But I keep hearing it, and it really stresses me out. Nevertheless, these comments from other people motivated me during the program.	Peer pressure		
The act of participating in the program alone made us more alert about taking care of our health. We were mindful about what to record, so we tried to do more exercise.	Available programs		
Even though there was a mountain right in front of my house, I didn’t go out to exercise. There were a plenty of places to exercise. Participating in the program developed my habit of exercising every morning.			
Messages like “You drank too much alcohol” made me become aware of my mistakes. I’d be like, “Oh, I may have indeed drunk a lot”. I once asked if drinking 500 cc of alcohol is considered too much (laughing). The feedbacks I get from time to time provide the opportunity to fix myself.	Experts’ advice		
The professor insisted that I needed to reduce my carbohydrate consumption. She told me to reduce it to two-thirds of the amount that I normally eat and to eat bland side dishes first. Eating bland side dishes first helped me eat less rice. Following her tips, I think I lost weight again.			
The step counter… Part of the reason why I started participating was because I wanted the step counter.	Decoys		
I wouldn’t call what I did an exercise, but whenever I had some free time during daily life, or whenever there was some gap time between one schedule and another, I would walk instead of taking the subway. I would walk around an hour this way.	Efforts (behavioral control) balancing and integrating with daily life	Internal reinforcement/autonomy	Lifestyle rebalancing under the self-regulation employing internal and external reinforcement
There’s a famous fish-shaped bun place right in front of my house. I used to buy 2000 won’s worth of buns whenever I passed by that place since they only give you three buns per 1000 won. Now, I don’t give into my temptation and just go my way.			
I forced myself to do what I didn’t want to do and walked 10,000 steps per week. I tried to walk over 10,000 steps within a week or walk at least one hour per day. Although I am doing the same thing over and over, it is hard to keep the habit of walking. I think being consistent is challenging. But over time, I slowly got used to walking.			
After losing weight by exercising, I felt myself looking better. So now, I have my own rule for losing weight. If I gain one kilogram, I control my eating. Using this method, I’ve been able to maintain my weight.			
I eat little even when I’m eating out. Even though I eat less, my weight on the scale keeps increasing. Since I’m forced to eat when I’m eating out with someone, I tend to avoid eating out with others. That was intriguing.			
I never thought I would dance. I had never done it before the program. I found myself enjoying it a lot and actually keeping up with it despite my age. I enjoyed it so much.	Finding fun		
I really like the music. They played the music we used to listen when we were students. I enjoyed losing the drastic weight I gained. I just enjoyed my life. Yeah, it was a lot of fun...			
Even on a hot summer day, even when it was raining, I made sure to walk on Wednesdays since Wednesdays were set as the “Walking Day”. On rainy days, the three of us would be walking in white raincoats (laughing). It was fun too. Walking was an extra source of fun aside from volunteering on Wednesdays. I had a lot of fun losing weight this summer.			
The reason why I am extending my participation is that I determined to increase my muscle mass for the next three months. I got curious about how much effort would be needed to gain how much muscle mass. Now that I can check my muscle mass on my own…	Continue with interest		
I already knew that my lifestyle was blamed to be fat. But when I checked one by one in this program, it seems clear that I was getting fat because of things that I should have changed.	Self-reflection		
Since I needed to fill out the checklist… I didn’t want to leave too many checkboxes empty, so I tried my best.	Self-monitoring		
It’s for me… It’s not for others. Even if I feel lazy, I still need to do it because it’s for myself. I think I did it just as how we live through our daily life.	Value/usefulness		
Seeing numerical improvements helped me understand my progress and motivated me to work harder.	Perceived benefits		
I was very fat in the beginning. I’m still fat now, and I was especially fat here (touching her armpits). One day, I shook my arms as I walked, and my arms felt so much lighter than it used to. They just felt light. At that point, I thought, “Oh, I think I am losing fat now”. But my weight on the scale still doesn’t change. Even then, I still enjoy doing it every day. I feel happy for myself.			
When I hiked five times a week, my health drastically became better. I lost weight, my disc conditions got better. My body just improved overall. It was really good.			
The fat around my waist… (laughing) I used to feel displeased whenever I looked at it in the washroom mirror, but it’s ok. I feel satisfied with myself now. It’s not like others can look at my progress, but I see the effect.			
I would exercise early in the morning, but some days, I feel tempted to just go home and sleep more. But once I beat that temptation and come back home after completing my exercise, I feel achieved and can start my day in a good mood.	Perceived competence		
I started out by walking. I noticed others were also walking... Once, a young person was jogging, not too fast, like this, and I tried to do the same, and it worked.			
After we began to actively walk, I got inspired by people around me who were walking to lose weight although I don’t know how motivated they were to walk. I think people around me had an influence on me. They motivated me to work harder.			
I enjoy walking, so walking worked the best for me. Everyone has a different method that works for them. To me, it was walking.	Preference/ Choice		
I started doing a “Wednesday walk” with other participants from here to Soongsil University. We would walk through the cemetery, and later the other two would walk toward their houses and I toward Singil-dong, which is where I live. We did this once every week together, and it helped a lot. We said we’d do this every Wednesday.	Peer supports	External reinforcement	
I asked a person who was also in the program how she did it. She did 30 sit-ups despite the pain. I felt motivated by her.			
Since I knew precise results would be out after a few months, I worked harder to achieve better results. When there are no accurate data to show my progress, I tend to slack off even if I was determined in the beginning. Now, I think about how my cholesterol levels would change in a blood test a few months later and am careful about what I eat.	Verifiable results		
Yes. I didn’t want to participate initially, but my daughter pushed me. She helps me a lot. I personally don’t enjoy exercising and I like eating. I told myself I’d give it my best try and I think I did this time. My health drastically got better. I’m thankful for my daughter. She still monitors what I eat.	Family supports		
I’d forget about the program and get reminded again by a text message. The fact that my coach still cared about me (laughing) motivated me to work harder.	Expert supports		
They show you how many kilometers you have walked so far at the Yangjae Stream. I can calculate how many calories I’ve burned by walking two kilometers.	Environmental stimulus		
You usually go to the gym to work out rather than just working out alone. Since you pay the gym to work out, you work out harder.	Economic efficacy		

**Table 3:** Inhibitors of healthy lifestyle modification and maintenance.

Inhibitors	Code	Subtheme	Theme
I ended up regaining weight from exercising less and eating on Thanksgiving.	Holiday event	Disruptions in daily routine	Failure to integrate healthy habits into lifestyle
I manage to control my appetite from Monday to Friday, and then end up eating again on Saturday and Sunday and regain weight. Even if I do well until Friday, if I end up drinking and eating out, my weight goes back to what it was, and this cycle keeps repeating. There are many occasions where I eat out with my family... and go out to have a drink with others, so it’s hard to stay consistent.	Eating out		
I was lying on the bed with a cast on my left leg which was injured. After that, two of my chest bones broke, so as soon as my leg healed, I had to stay on the bed for another four to six weeks. Then I broke my right leg. I ended up eating snacks a lot since I spent all my time at home. I’d wallow around and keep opening and closing the fridge. I think eating snacks became a habit because of this experience.	Injury		
I also end up breaking the rules when I’m traveling.	Travel		
The stress that accumulated over the course of the day… I also feed my kids all three meals and end up eating with them. I get tired from work and from feeding my kids. I think I tried to cope with the stress by eating.	Bad habits related to daily life stress	Decreased autonomy	
I spend a lot of my time with kids, and I love eating. I can’t help but eat some of my kids’ snacks… I think I felt stressed about having my alone time taken away by the kids and tried to cope with the stress by eating, which resulted in my weight gain.			
When you become obsessed… You’re supposed to enjoy it. You tell yourself you have to do something, and that makes it even harder.	Pressure/ tension related to the program		
To me, the pressure to keep up was very stressful. I felt like if I didn’t keep up, I would fall behind everyone else. Having to fill out the self-checklist on the computer according to the schedule was too much for me, so I just gave up.			
I feel so pathetic and depressed. I would try but fail. I would see how weak-willed I am and feel unenergetic. And then again, I would tell myself to try again. Even then, I’d still feel unenergetic for a long time. I’m so pathetic.	Lowered perceptions of competence		
I keep telling myself I’ll start today or tomorrow. I feel so pathetic and feel frustrated with me. My head knows what’s right, but once I see it, I can’t help it. I’d regret and tell myself I shouldn’t have eaten, but I keep making the same mistakes.			
I love sweets. My head tells me not to eat them, but once I see them, I can’t help it. I think that’s the problem.	Lack of perceived eating control		
I love flour. I am a fan of bread. I know it’s not good for you, but my desire for it is stronger. Even if I’m full, I’d eat bread if there’s one. One person told me I’d lose 2–3 kilos by simply cutting off bread. What can I do, though? My desire for bread is just too strong. If I had won during that fight, I would not weight over 50 kg right now.			
I do think that I need to work out, but the busy schedule I set for myself keeps me from working out.	Deprioritize		
I don’t know if it’s because I don’t see any immediate threats to my health, but I keep skipping exercise if I feel a bit tired.	Lack of perceived threat		
My thyroid conditions make me feel weak and lethargic. I keep skipping exercises by making excuses about my thyroid conditions. It’s actually difficult for me to move. You know what I mean… I feel very tired and crave sweets. I gained two kilograms as a result, and it’s been hard to lose the gained weight	physical limitations		
I used to exercise a lot initially. I even walked around Santiago and lost 3 kg but regained the weight in a week. I haven’t seen any change in my weight, not even 0.1 kg loss. There’s no change at all no matter how much I exercise.	Unmet expectations	Insufficient reinforcement	Inappropriate supportive strategy
I did eat out a lot, but I was hopeful that my health level would improve. They turned out to have worsened, and it really disappointed me. I felt so discouraged. Plus, my knees have also been hurting. In the past, I used to climb the stairs even when I was tired and enjoyed doing it, but lately, I’ve been careful with exercising.			
You know how you feel when your results are not up to your expectations. When we were young, we would eat a lot but if you exercise one day, you’d see the effect of it the next day. Now, even after three or four days, postmenopausal women like me don’t see any improvements because we’re old. Since we’re not seeing immediate improvements, it’s harder to maintain a healthy lifestyle. Seeing even just a small improvement would motivate us but without seeing any improvement, we can’t get ourselves to it.			
I did a blood test only to find out that my sugar levels hadn’t dropped and felt like this wasn’t working either... I just ended up getting lazier and inconsistent.			
I was hopeful that since I was doing something that I never used to do and I was working hard, I would see drastic improvements, but that was not the case… and it made me skeptical. Since there were no immediate improvements…			
I used to go to the gym to use the bike machine and run on the treadmill, but the air inside the gym felt so suffocating. I ended up not going to the gym often…	Environmental limitations		
When it’s raining heavily, I don’t go out. When there’s no one outside, it’s scary. The Han River is bustling with people even after midnight. At the Ttukseom Park, you can go out any time to exercise, but even there, it’s scary when it’s rainy and dark with no people around…			
The internal medicine doctor didn’t tell me to lose weight contrary to what I expected. I found that a bit strange, but it did make me slack off a bit.	Lack of experts advise		
I think the program was a bit ineffective because they didn’t tell me to walk exactly how many kilometers.	Deficit of precise directions		
I didn’t want to hike alone, so I just didn’t go hiking. My blood pressure started rising again after I stopped hiking	Lack of supportive relationships		
I had fun exercising during the program, but I couldn’t get myself to do the same exercise alone at home.			
What I really hate is when a friend brings brown rice to our hangout session because she’s on a diet. I hate that! She shouldn’t have come! Her doing that really breaks the mood. I get that she’s strong-willed and may later be successful with weight loss, but I let myself eat when we’re hanging out. I guess it’s just different opinions, but people like her ruin my mood. How can you bring a lunch like that? I just let myself loose when we’re hanging out and eat to my heart’s content. That’s why I have all this fat.	Perceived norms	Subjective norms	
I felt so weak after I lost around one kilogram. I told my friends I felt weak, and my friends told me, “Hey, it’s because you lost weight. Just eat” (laughing). They told me that people who eat well look well, and that when you lose weight at our age, you are bound to feel weak.			
You’re throwing away the effort of the person who cooked the meal when you’re throwing away a meal. You know they say you need to look good when you die (laughing), so you have to eat.			
I’d just accept one drink and drink maybe half of it just to go along with the mood.			

## References

[1] El Khoudary S.R., Greendale G., Crawford S.L., Avis N.E., Brooks M.M., Thurston R.C., Karvonen-Gutierrez C., Waetjen L.E., Matthews K. (2019). The menopause transition and women’s health at midlife: A progress report from the study of women’s health across the nation (swan). Menopause.

[2] Im E.O., Choe M.A. (2004). Korean women’s attitudes toward physical activity. Res. Nurs. Health.

[3] Shufelt C.L., Pacheco C., Tweet M.S., Miller V.M. (2018). Sex-specific physiology and cardiovascular disease. Adv. Exp. Med. Biol..

[4] Garcia M., Mulvagh S.L., Merz C.N.B., Buring J.E., Manson J.E. (2016). Cardiovascular disease in women: Clinical perspectives. Circ. Res..

[5] Sun Y., Liu B., Snetselaar L.G., Wallace R.B., Caan B.J., Rohan T.E., Neuhouser M.L., Shadyab A.H., Chlebowski R.T., Manson J.E. (2019). Association of normal-weight central obesity with all-cause and cause-specific mortality among postmenopausal women. JAMA Netw. Open.

[6] Renehan A.G., Howell A. (2005). Preventing cancer, cardiovascular disease, and diabetes. Lancet.

[7] Taubert K.A., Clark N.G., Smith R.A. (2007). Patient-centered prevention strategies for cardiovascular disease, cancer and diabetes. Nat. Clin. Pract. Cardiovasc. Med..

[8] Kapoor E., Collazo-Clavell M.L., Faubion S.S. (2017). Weight gain in women at midlife: A concise review of the pathophysiology and strategies for management. Mayo Clin. Proc..

[9] Karimlou V., Charandabi S.M.-A., Malakouti J., Mirghafourvand M. (2019). Effect of counselling on health-promoting lifestyle and the quality of life in iranian middle-aged women: A randomised controlled clinical trial. BMC Health Serv. Res..

[10] Neumann A., Lindholm L., Norberg M., Schoffer O., Klug S.J., Norström F. (2017). The cost-effectiveness of interventions targeting lifestyle change for the prevention of diabetes in a swedish primary care and community based prevention program. Eur. J. Health Econ..

[11] Mahdipour N., Shahnazi H., Hassanzadeh A., Sharifirad G. (2015). The effect of educational intervention on health promoting lifestyle: Focusing on middle-aged women. J. Educ. Health Promot..

[12] Kim H.R., Kim H.S. (2017). Autonomy-supportive, web-based lifestyle modification for cardiometabolic risk in postmenopausal women: Randomized trial. Nurs. Health Sci..

[13] Kreidieh D., Itani L., El Kassas G., El Masri D., Calugi S., Dalle Grave R., El Ghoch M. (2018). Long-term lifestyle-modification programs for overweight and obesity management in the arab states: Systematic review and meta-analysis. Curr. Diabetes Rev..

[14] Tollosa D.N., Holliday E., Hure A., Tavener M., James E.L. (2020). A 15-year follow-up study on long-term adherence to health behaviour recommendations in women diagnosed with breast cancer. Breast Cancer Res. Treat..

[15] Hosseini H., Moradi R., Kazemi A., Shahshahani M.S. (2017). Determinants of physical activity in middle-aged woman in isfahan using the health belief model. J. Educ. Health Promot..

[16] Ehlers D.K., Huberty J.L. (2014). Middle-aged women’s preferred theory-based features in mobile physical activity applications. J. Phys. Act. Health.

[17] Vallance J.K., Murray T.C., Johnson S.T., Elavsky S. (2011). Understanding physical activity intentions and behavior in postmenopausal women: An application of the theory of planned behavior. Int. J. Behav. Med..

[18] Ban H.-J., Kim H.-S. (2020). Applying the modified health belief model (HBM) to korean medical tourism. Int. J. Environ. Res. Public Health.

[19] Wang C.J., Liu W.C., Kee Y.H., Chian L.K. (2019). Competence, autonomy, and relatedness in the classroom: Understanding students’ motivational processes using the self-determination theory. Heliyon.

[20] Si H., Shi J.-G., Tang D., Wen S., Miao W., Duan K. (2019). Application of the theory of planned behavior in environmental science: A comprehensive bibliometric analysis. Int. J. Environ. Res. Public Health.

[21] Oyibo K., Adaji I., Vassileva J. (2018). Social cognitive determinants of exercise behavior in the context of behavior modeling: A mixed method approach. Digit. Health.

[22] Kang Y.S., Chung M.J., Park Y.S., Lee Y.S., Kim H.S., Lee D.M., Lee D.W. (2009). An analysis of articles for health promotion behaviors of korean middle-aged. J. Korean Acad. Commun. Health Nurs..

[23] Annesi J.J. (2017). Mediation of the relationship of behavioural treatment type and changes in psychological predictors of healthy eating by body satisfaction changes in women with obesity. Obes. Res. Clin. Pract..

[24] Mohammadi Zeidi B., Kariman N., Kashi Z., Mohammadi Zeidi I., Alavi Majd H. (2020). Predictors of physical activity following gestational diabetes: Application of health action process approach. Nurs. Open.

[25] Metzgar C.J., Preston A.G., Miller D.L., Nickols-Richardson S.M. (2015). Facilitators and barriers to weight loss and weight loss maintenance: A qualitative exploration. J. Hum. Nutr. Diet.

[26] Samdal G.B., Eide G.E., Barth T., Williams G., Meland E. (2017). Effective behaviour change techniques for physical activity and healthy eating in overweight and obese adults; systematic review and meta-regression analyses. Int. J. Behav. Nutr. Phys. Act..

[27] Barragan R., Rubio L., Portoles O., Asensio E.M., Ortega C., Sorlí J.V., Corella D. (2018). Qualitative study of the differences between men and women’s perception of obesity, its causes, tackling and repercussions on health. Nutr. Hosp..

[28] Walker K.C., Valentiner L.S., Langberg H. (2018). Motivational factors for initiating, implementing, and maintaining physical activity behavior following a rehabilitation program for patients with type 2 diabetes: A longitudinal, qualitative, interview study. Patient Prefer. Adherence.

[29] Sevild C.H., Niemiec C.P., Bru L.E., Dyrstad S.M., Husebø A.M.L. (2020). Initiation and maintenance of lifestyle changes among participants in a healthy life centre: A qualitative study. BMC Public Health.

[30] Khandelwal S. (2020). Obesity in midlifestyle and dietary strategies. Clinacterics.

[31] Bauman A., Merom D., Bull F.C., Buchner D.M., Fiatarone Singh M.A. (2016). Updating the evidence for physical activity: Summative reviews of the epidemiological evidence, prevalence, and interventions to promote “active aging”. Gerontologist.

[32] Lemamsha H., Papadopoulos C., Randhawa G. (2018). Understanding the risk and protective factors associated with obesity amongst libyan adults-a qualitative study. BMC Public Health.

[33] Poirier N., Légaré F., Stacey D., Lemieux S., Bégin C., Lapointe A., Desroches S. (2016). Postmenopausal women with abdominal obesity choosing a nutritional approach for weight loss: A decisional needs assessment. Maturitas.

[34] Young S.D. (2020). The adaptive behavioral components (ABC) model for planning longitudinal behavioral technology-based health interventions: A theoretical framework. J. Med. Int. Res..

[35] Kwasnicka D., Dombrowski S.U., White M., Sniehotta F. (2016). Theoretical explanations for maintenance of behaviour change: A systematic review of behaviour theories. Health Psychol. Rev..

[36] Dunton G.F., Rothman A.J., Leventhal A.M., Intille S.S. (2019). How intensive longitudinal data can stimulate advances in health behavior maintenance theories and interventions. Transl. Behav. Med..

